# RRM2B Is Frequently Amplified Across Multiple Tumor Types: Implications for DNA Repair, Cellular Survival, and Cancer Therapy

**DOI:** 10.3389/fgene.2021.628758

**Published:** 2021-03-12

**Authors:** Waleed Iqbal, Elena V. Demidova, Samantha Serrao, Taha ValizadehAslani, Gail Rosen, Sanjeevani Arora

**Affiliations:** ^1^Cancer Prevention and Control Program, Fox Chase Cancer Center, Philadelphia, PA, United States; ^2^School of Biomedical Engineering, Science and Health Systems, Drexel University College of Engineering, Philadelphia, PA, United States; ^3^Institute of Fundamental Medicine and Biology, Kazan Federal University, Kazan, Russia; ^4^Department of Epidemiology and Biostatistics, Dornsife School of Public Health, Drexel University, Philadelphia, PA, United States; ^5^Department of Electrical and Computer Engineering, College of Engineering, Drexel University, Philadelphia, PA, United States; ^6^Department of Radiation Oncology, Fox Chase Cancer Center, Philadelphia, PA, United States

**Keywords:** chromosome 8, 8q-amplicon, *RRM2B*, cancer, *MYC*

## Abstract

*RRM2B* plays a crucial role in DNA replication, repair and oxidative stress. While germline *RRM2B* mutations have been implicated in mitochondrial disorders, its relevance to cancer has not been established. Here, using TCGA studies, we investigated *RRM2B* alterations in cancer. We found that *RRM2B* is highly amplified in multiple tumor types, particularly in *MYC*-amplified tumors, and is associated with increased *RRM2B* mRNA expression. We also observed that the chromosomal region 8q22.3–8q24, is amplified in multiple tumors, and includes *RRM2B*, *MYC* along with several other cancer-associated genes. An analysis of genes within this 8q-amplicon showed that cancers that have both *RRM2B*-amplified along with *MYC* have a distinct pattern of amplification compared to cancers that are unaltered or those that have amplifications in *RRM2B* or *MYC* only. Investigation of curated biological interactions revealed that gene products of the amplified 8q22.3–8q24 region have important roles in DNA repair, DNA damage response, oxygen sensing, and apoptosis pathways and interact functionally. Notably, *RRM2B*-amplified cancers are characterized by mutation signatures of defective DNA repair and oxidative stress, and at least *RRM2B*-amplified breast cancers are associated with poor clinical outcome. These data suggest alterations in RR2MB and possibly the interacting 8q-proteins could have a profound effect on regulatory pathways such as DNA repair and cellular survival, highlighting therapeutic opportunities in these cancers.

## Introduction

RRM2B plays an important role in regulating replication stress, DNA damage, and genomic stability (Aye et al., 2015; Foskolou et al., 2017). *RRM2B* encodes a small subunit of p53-inducible ribonucleotide reductase (RNR). The RNR is a heterotetrametric enzyme responsible for the *de novo* conversion of ribonucleotide diphosphates into the corresponding deoxyribonucleotide diphosphates for DNA synthesis, thus playing an important role in maintaining deoxyribonucleotide pools (Okumura et al., 2005). The large subunit of the RNR complex consists of a dimer of the RRM1 protein, while the small subunit dimer is either RRM2 or RRM2B (varies depending on cellular conditions). P53-dependent induction of RRM2B expression by hypoxia leads to the exchange of the small RNR subunit from RRM2 to RRM2B, forming a new RNR complex that drives basal DNA replication, reduces replication stress, and maintains genomic stability (Wang et al., 2011; Foskolou and Hammond, 2017). These known functions of RRM2B suggest that *RRM2B* alterations may play a role in tumorigenesis (Aye et al., 2015; Foskolou et al., 2017).

RRM2B is located on chromosome 8q [8q23.1 (Tanaka et al., 2000); in 2018, annotation changed to 8q22.3]^1^. Germline missense and loss of function mutations in *RRM2B* have been associated with mitochondrial depletion syndrome (MDS), with distinct but variable clinical phenotype (Gorman and Taylor, 1993; Bornstein et al., 2008). At present, there are no known *RRM2B* germline alterations associated with cancer risk. However, somatic changes in *RRM2B*, including most typically amplifications, have been observed in breast, liver, lung and skin cancers (Chae et al., 2016). In a survey of the COSMIC database, *RRM2B* emerged as the most highly amplified DNA repair gene (Chae et al., 2016). Additionally, TCGA studies of ovarian, breast, liver, and prostate cancer, have found that cases with *RRM2B* copy number variations (CNV) (amplifications and deletions) have decreased overall survival (OS) (Chae et al., 2016). Similarly, increased metastasis and poor prognosis were correlated with *RRM2B* overexpression in head and neck cancer (Yanamoto et al., 2003), esophageal cancer (Okumura et al., 2006) and lung sarcomatoid carcinoma (Chen et al., 2017). Another study noted that elevated expression of *RRM2B* is associated with better survival in advanced colorectal cancer (Liu et al., 2011).

*RRM2B* amplification may be driven by selection of *RRM2B* function, or *RRM2B* may be amplified as a passenger, concurrent with selection for a nearby gene with a driver activity in cancer. In breast cancer, multiple genes localized in the 8q12.1–8q24.23 interval were found to be amplified, including *RRM2B* (Parris et al., 2014). Most *RRM2B* amplifications are accompanied by *MYC* amplifications, and these two genes are located in close proximity (Christoph et al., 1999). However, *RRM2B* amplifications also occur independent of *MYC* amplifications, albeit at a lower frequency (Kalkat et al., 2017). While multiple studies have observed the amplification of the 8q region, currently the frequency and specificity of these amplifications is not known, and more specifically, the consequence of *RRM2B* amplification with *MYC* or as independent from *MYC* amplification is not clearly understood (Bruch et al., 1998; Sato et al., 1999; Saha et al., 2001; Byrne et al., 2005; Ehlers et al., 2005; Ho et al., 2006; Schleicher et al., 2007; Bilal et al., 2012; Parris et al., 2014; Yong et al., 2014; Kwon et al., 2017).

Here, using data from TCGA, we found that *RRM2B*-amplified tumors not only exhibit increased *RRM2B* expression in multiple cancers (such as breast, ovarian, head, and neck cancer), but also exhibit distinct mutation signatures. Analysis of the most common breast cancer subtype indicated that *RRM2B* amplifications may independently impact clinical outcomes in these cancers. Further, tumors bearing *RRM2B* amplifications showed that several genes in the 8q22–8q24 region (along with *MYC*) are highly amplified. Additionally, analysis of 8q-proteins suggests functional interaction within the same cell regulatory mechanisms of DNA damage response and repair, hypoxia and apoptosis. Based on these results, we hypothesize that while *MYC* may be the cancer driver, co-amplification of *RRM2B* and other 8q-genes may be relevant for cancer cell survival. These finding suggest opportunities for novel therapeutic targeting strategies (such as those targeting DNA damage response and repair) for tumors carrying *RRM2B* alterations.

## Materials and Methods

### Analysis of Alteration Frequencies Using TCGA Studies

TCGA studies were accessed using the cBioPortal website^2^ (Cerami et al., 2012). Data was downloaded (on 6/21/2018) from 30 TCGA studies for tumors that have been profiled for mutations as well as copy number variants. For cancer types with the highest frequency of *RRM2B* amplifications, cases were segregated based on co-occurrence of *TP53* mutations or *MYC* amplifications. The *TP53* alterations were all mutations (missense, and truncating). The truncating mutations in TCGA are frameshift deletions, frameshift insertions, nonsense, splice site. All mutations were analyzed for co-occurrence, including those that were homozygous or heterozygous.

### RNA Expression

RNA seq. V2 RSEM data was downloaded from OV (ovarian cancer study), BRCA (breast cancer study) and HNSC (head and neck cancer study) studies on 11/7/2018, and 3/19/2020. The RNA seq. data were only analyzed for tumors that were also profiled for copy number variants. Cases were segregated based on copy number variants type: deep deletion, shallow deletion, diploid, gain and amplification. For *RRM2B* there was only a single case of deep deletion out of all (*n* = 2,181 cases from all studies), observed in BRCA, and was removed. For *CCNE2*, *EI3FE*, *MTDH, MYC, RAD21, TP53INP1*, and *YWHAZ*—there were no cases of deep deletion for any of the genes in the HNSC study, but there was one case each in *EI3FE*, *RAD21* and *YWHAZ* with deep deletions in the BRCA study, and also one case each in *EI3FE* and *MTDH* as well as two cases in *RAD21* for the OV study. V2 RSEM from cases with different copy number variants categories and statistical significance was tested in Graphpad Prism 8.0 using Mann-Whitney Non-Parametric *T*-test (Gao and Song, 2005; Li and Tibshirani, 2013). Additionally, plots of log2 mRNA expression values based on relative linear copy number values were used to test for correlation between increasing copy number change and mRNA expression, then Pearson coefficient (Karl and Francis, 1997) and log rank (Mantel, 1966) p-values for each cancer type were calculated. Graphpad Prism 8.0 was used for all statistical analysis. For *CCNE2, EI3FE, MTDH, MYC, RAD21, TP53INP1* and *YWHAZ*: the data were analyzed as above but are presented as Supplementary Tables with expression data and statistics.

### Tumor Mutation Signatures

Tumor whole-exome sequence data in the TCGA Pan-Cancer Atlas studies (for OV, BRCA, HNSC, and LUAD), that includes spectra of individual tumors, was downloaded from the Synapse platform^3^ on 11/5/2018. Next, IDs of subjects with different cancer types were downloaded from the cBioPortal website^2^, and subjects with amplification in *RRM2B* and *MYC* genes were selected. Presence or absence of these amplifications classified the subjects into four categories: (1) cases with amplification in both *RRM2B* and *MYC*, (2) cases with amplification in *RRM2B*, but not in *MYC*, (3) cases with amplification in *MYC*, but not in *RRM2B*, (4) and cases without amplification in both genes. For each specific cancer type, mutational spectra of each category were calculated by finding the average number of amplifications in each of the 96 mutation classes. To compare differences in these 96 mutation types, one-way ANOVA (Welch, 1951), two-way ANOVA (Fujikoshi, 1993; Shuster et al., 2008), and Wilcoxon rank-sum test (Shuster et al., 2008) were used to test for significant differences. In order to eliminate the possibility of false discoveries caused by multiple comparison, in each test, Benjamini–Hochberg correction (Benjamini and Hochberg, 1995) was applied to each group of 96 p-values (corresponding to 96 mutations). For each cancer type the patients were divided into the following mutation signature groups: (1) cases with amplification in both *RRM2B* and *MYC*, (2) cases with amplification in *RRM2B*, but not in *MYC*, (3) cases with amplification in *MYC*, but not in *RRM2B*, (4) and cases without amplification in both genes, and anova1, anova2 and Wilcoxon rank-sum functions in MATLAB R2018a were used to test for significance. Each signature type was determined to be significant only if its *P*-value was below 0.05 in the one-way ANOVA test. The two-way ANOVA was used to distinguish the impact of factors such as co-amplifications with *MYC*. Additionally, the Wilcoxon rank-sum test was also performed as a secondary method to test for significance.

### Clinical Outcomes

Clinical data was downloaded for the breast cancer (BRCA) TCGA study. Patients were divided into different groups based on having amplifications in *RRM2B* or *MYC*, in both or neither. The clinical data from each group were used to generate OS and disease/progression-free survival (DFS) Kaplan-Meier curves (Kaplan and Meier, 1958) using GraphPad Prism 8.0, and each curve was compared with the other respective curves using the log-rank test (Mantel, 1966) and significance was shown based on ^∗^*P* < 0.05, ^∗∗^*P* < 0.01, and ^∗∗∗^*P* < 0.001. The significance level was set at *p* < 0.05. Additionally, Wilcoxon tests were also used (Hazra and Gogtay, 2017), and significance was shown based on ^∗^*P* < 0.05, ^∗∗^*P* < 0.01, and ^∗∗∗^*P* < 0.001. The significance level was set at *p* < 0.05.

### Analysis of Amplifications in 8q-Genes

The tumors that were assessed for alteration frequency were also analyzed for amplifications in the 8q-genes. Tumor data was downloaded on 6/22/18 from cbioportal.org. The genes used for analysis are located on chromosome 8 from the region 8q11.2–8q24.3 and have been associated with cancer according to the Cancer Index^4^. The amplification pattern of other 8q-genes in BC, OC, and HNSCC was compared to cases with co-amplifications of *MYC* and *RRM2B*, cases with single amplifications or cases that were unaltered. A univariate analysis was conducted to calculate the positive or negative linear trend for the frequency of gene amplification, assessed at 95% confidence interval. The Pearson correlation coefficient and *p*-values were computed by using PROC CORR in SAS 9.4. The significance level was set at *p* < 0.05.

### Protein Interaction and Pathway Enrichment

Data for the RRM2B interaction network was downloaded from the BioGRID database (Oughtred et al., 2019) (10/25/2018) and analyzed by Cytoscape v. 3.6.1 (Shannon et al., 2003). Several interactions were added manually based on literature findings [RRM2B-FOXO3 (Cho et al., 2014), RRM2B-P21 (Xue et al., 2007), RRM2B-TP73 (Tebbi et al., 2015), RRM2B-E2F1 (Qi et al., 2015), RRM2B-MEK2 (Piao et al., 2012)]. WebGestalt (WEB-based GEne SeT AnaLysis Toolkit) was used for gene set enrichment analysis to extract biological insights from the genes of interest (Liao et al., 2019). The online WebGestalt tool was used, and an over representation analysis was performed. The enrichment results were prioritized by significant *p*-values, and FDR thresholds at 0.01.

## Results

### *RRM2B* Is Highly Amplified in Multiple Cancers, With an Amplification Frequency Similar to *MYC*, While Alterations in Other RNR Genes Are Infrequent

**FIGURE 1:**
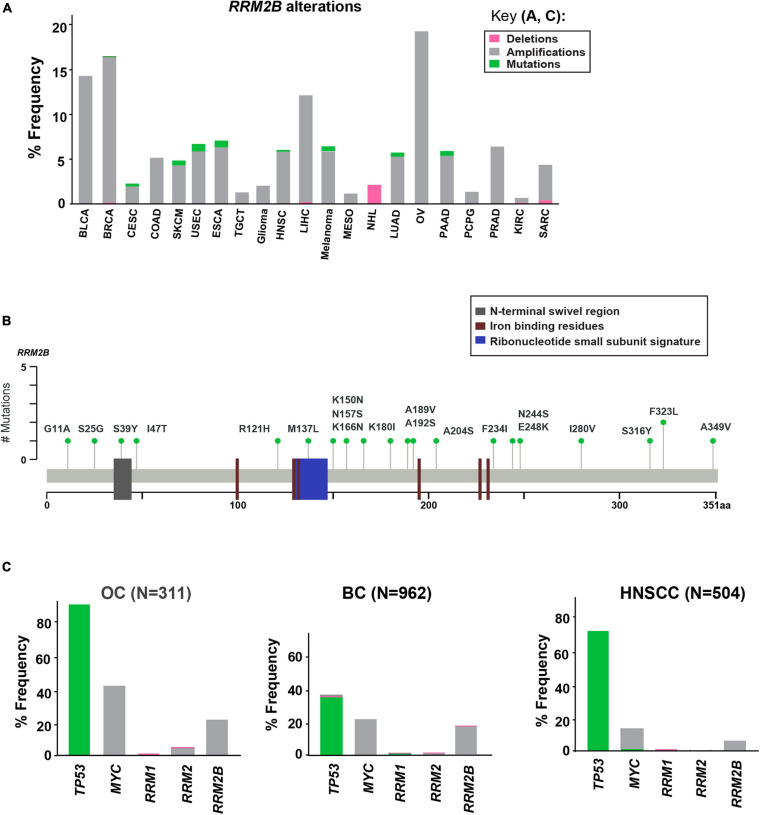
Somatic alterations in *RRM2B* from cbioportal.org. **(A)** Somatic alterations in *RRM2B* across TCGA studies. BLCA, Bladder urothelial carcinoma; BRCA, Breast invasive carcinoma; CESC, Cervical squamous cell carcinoma and endocervical adenocarcinoma; COAD, Colon adenocarcinoma; SKCM, Skin cutaneous melanoma; USEC, Uterine corpus endometrial carcinoma; ESCA, Esophageal carcinoma; TGCT, Testicular germ cell tumors; HNSC, Head and neck squamous cell carcinoma; LIHC, Liver hepatocellular carcinoma; MESO, Mesothelioma; NHL, Non-Hodgkin lymphoma; LUAD, Lung adenocarcinoma; OV, Ovarian serous cystadenocarcinoma; PAAD, Pancreatic adenocarcinoma; PCPG, Pheochromocytoma and paraganglioma; PRAD, Prostate adenocarcinoma; KIRC, Kidney renal clear cell carcinoma; SARC, Sarcoma. Amplifications (gray), mutations (green), and deletions (pink) are represented as percent frequency. **(B)** *RRM2B* mutations in TCGA. 2D RRM2B protein stick figure showing the important domains of RRM2B [N-terminal swivel region, required for dimer stability, gray; ribonucleotide small subunit signature (conserved region in catalytic site between RRM2 and RRM2B), blue, and iron-binding residues required for catalytic activity, red] and the number of somatic mutations in RRM2B. **(C)** Frequency of *TP53*, *MYC*, *RRM1*, *RRM2* and *RRM2B* alterations in ovarian (OC), breast (BC), and head and neck (HNSCC) cases.

Using cancer cases from TCGA, we observed that *RRM2B* is frequently amplified, with the highest percentage observed in ovarian, breast, bladder, and liver cancers (21.54–14.5%), and a lower rate of amplifications in multiple other cancers (14–0.6%) (Figure 1A). Deletions of *RRM2B* were only observed in Non-Hodgkin’s lymphoma (∼2%). In addition to amplifications, a low frequency of mutations (<2%) were observed in head and neck, lung, endometrial, esophagogastric, cervical, and pancreatic cancers (Figure 1A). Mapping of somatic *RRM2B* mutations to the RRM2B protein structure indicated the mutations are present in multiple regions of the protein and do not always cluster at defined functional domains/regions (Smith et al., 2009; Maatta et al., 2016; Finsterer and Zarrouk-Mahjoub, 2018), unlike the mutations observed in mitochondrial disorders, which typically result in reduced or eliminated function of the RRM2B protein (Gorman and Taylor, 1993; Bornstein et al., 2008; Figure 1B). Only the R121H mutation found in this study has been previously observed in a patient with mitochondrial neurogastrointestinal encephalopathy (Gorman and Taylor, 1993; Shaibani et al., 2009). This mutation has been predicted to impact the docking interface of the ribonucleoside reductase small subunit homodimer and thereby impact protein activity.

Since RRM2B is regulated by TP53 and is typically co-amplified with *MYC* (Figure 1), we next compared the frequency of *RRM2B* alterations with alterations in *TP53*, and *MYC*. We also compared the frequency of *RRM2B* alterations along with the members of the RNR complex [*RRM1*, *RRM2* (Kolberg et al., 2004; Figure 1C)]. For this analysis, we selected breast and ovarian cancer studies as they had the highest frequency of *RRM2B* alterations. While head and neck cancers carried a lower frequency of amplifications in *RRM2B* (similar to few other tumor types in Figure 1A), we chose this tumor type because of the clinical significance of *TP53* alteration status to HNSCCs (Zhou et al., 2016) and the known regulation of RRM2B by p53. As expected, *TP53* was the most altered gene in the studied cancers, however, *RRM2B* amplifications did not always significantly co-occur with *TP53*. Interestingly, most cases with *TP53* mutations (which were missense, and truncating mutations) did not have *RRM2B* amplifications (Supplementary Figure 1A). In comparison, a greater number of cases with *MYC* amplifications were observed to also have *RRM2B* co-amplifications. For ovarian (OC) and head and neck squamous cell carcinoma (HNSCC), this was half of all cases with *MYC* amplifications, while in breast cancer (BC) this was ∼90% cases (Supplementary Figure 1B). Finally, *RRM1* and *RRM2*, the functional partners of RRM2B, were infrequently altered and did not co-occur with *RRM2B* amplifications (Figure 1C).

### Tumors With *RRM2B* Amplifications Exhibit Increased *RRM2B* Expression

Previous studies have not shown whether tumors carrying amplified *RRM2B* have increased RRM2B expression, which might directly impact its role in cancer. Multiple studies have observed that gene amplifications do not always lead to increased expression (Jia et al., 2016). Thus, to confirm increased expression, we used mRNA expression data from three TCGA studies (OV, BRCA, HNSC), and found that tumors with gains and amplifications had significantly increased *RRM2B* mRNA expression in all three cancer types studied (Supplementary Figure 2). *RRM2B* amplifications also significantly correlated with an increase in *RRM2B* mRNA expression in OC and BC, and with an increased trend in HNSCC (OC: Pearson correlation = 0.64, Log rank *p-*value = 0.05; BC: Pearson correlation = 0.53, Log rank *p*-value = 0.04; HNSCC: Pearson correlation = 0.32, Log rank *p-*value = 0.08) (Supplementary Figure 2).

### *RRM2B* Amplification by Itself or Co-amplification With *MYC* Is in an 8q-Amplicon That Is Present in Multiple Cancer Types

**FIGURE 2:**
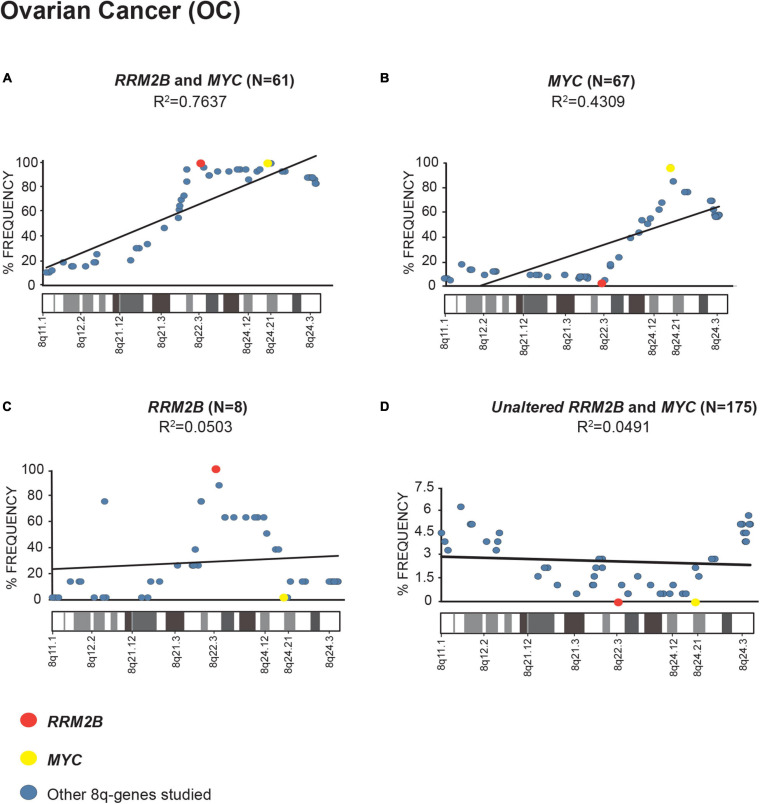
Amplification frequency of 8q-genes in ovarian cancer (OC). **(A)** cases with co-amplification of *RRM2B* and *MYC*; **(B)** cases *MYC* only amplification; **(C)** cases *RRM2B* only amplifications; **(D)** cases with neither (unaltered) were plotted as percent of frequency for amplifications in various 8q-region genes relevant for cancer (see Supplementary Table 1). *RRM2B* (red circle), *MYC* (yellow circle), and other genes (blue circle). The Pearson correlation (*R*^2^ value) for the data points is represented by a black trend line.

In multiple cancers, we identified that *RRM2B* and/or *MYC* were amplified as part of an amplicon with multiple other 8q-genes (Supplementary Table 1, list of cancer relevant genes and gene ontology classification). We queried the amplifications in OC (Figure 2), BC (Supplementary Figure 3), and HNSCC (Supplementary Figure 4) cancers in TCGA.

We observed a strong Pearson correlation for amplifications in the 8q11–8q24 region in cases with *RRM2B* and *MYC* co-amplifications. The strongest correlation was observed for OC (*R*^2^ = 0.7637, *p* < 0.00001, Figure 2A). The cases with either *MYC* or *RRM2B* amplification alone (*R*^2^ = 0.4309, *p* = 0.000273, Figure 2B, and *R*^2^ = 0.0.0503, *p* = 0.905, Figure 2C) or those without (*R*^2^ = 0.7637, *p* = 0.518, Figure 2D) showed weaker Pearson correlation. These amplifications observed (Figures 2A–C) are in the chromosome segment between *RRM2B* (8q22.3) and *MYC* (8q24.21). This amplicon contains 11 cancer relevant genes: *BALC, ANGPT1, EIF3E, EBAG9, TRSP1, RAD21, EXT1, TNFRSF11B, NOV, HAS2*, and *RNF139* (Supplementary Table 1).

**FIGURE 3:**
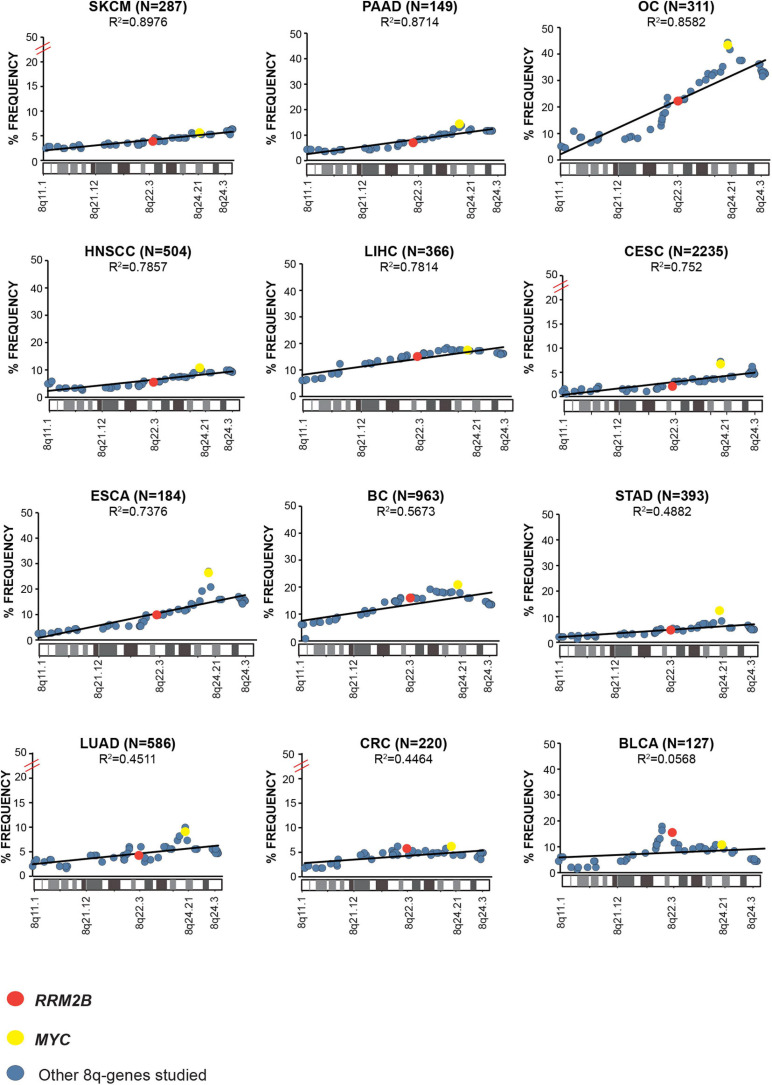
Amplification frequency of cancer relevant 8q-genes in SKCM, Skin cutaneous melanoma; PAAD, Pancreatic adenocarcinoma; OC, Ovarian serous cystadenocarcinoma; HNSCC, Head and neck squamous cell carcinoma; LIHC, Liver hepatocellular carcinoma; CESC, Cervical and endocervical cancers; ESCA, Esophageal carcinoma; BC, Breast invasive carcinoma; STAD, Stomach adenocarcinoma; LUAD, Lung adenocarcinoma; COAD, Colon adenocarcinoma; BLCA, Bladder urothelial carcinoma, amplifications were plotted as percent of frequency for amplifications. *RRM2B* (red circle), *MYC* (yellow circle), and other genes (blue circle). The Pearson correlation (*R*^2^ value) for the data points is represented by a black trend line.

Next, we examined the overall amplification frequency of 8q genes using TCGA data, without segregating cases based on *RRM2B* and/or *MYC* amplifications. We found, that the 8q11.3–8q24 amplicon is present in multiple tumor types (Figure 3). A positive correlation was observed for increased amplifications in the 8q11–8q24 region, which includes *RRM2B* and *MYC* (Pearson correlation range: *R*^2^ = 0.8976–0.0568). The strongest correlations were observed for skin (SKCM, *R*^2^ = 0.8976, *p* < 0.00001), pancreatic adenocarcinoma (PAAD, *R*^2^ = 0.8714, *p* < 0.00001), ovarian cancer (OV, *R*^2^ = 0.8582, *p* < 0.00001), liver hepatocellular carcinoma (LIHC, *R*^2^ = 0.7814, *p* < 0.00001), and esophageal cancer (ESCA, *R*^2^ = 0.7376, *p* < 0.00001) (Figure 3). All other correlations were significant at *p* < 0.00001, except for BLCA (*p* = 0.525). Finally, we also observed that mRNA expression of multiple genes in the amplicon was also significantly increased (Supplementary Table 2).

### *RRM2B* Protein Interaction Network Includes Co-amplified 8q-Amplicon Genes

**FIGURE 4:**
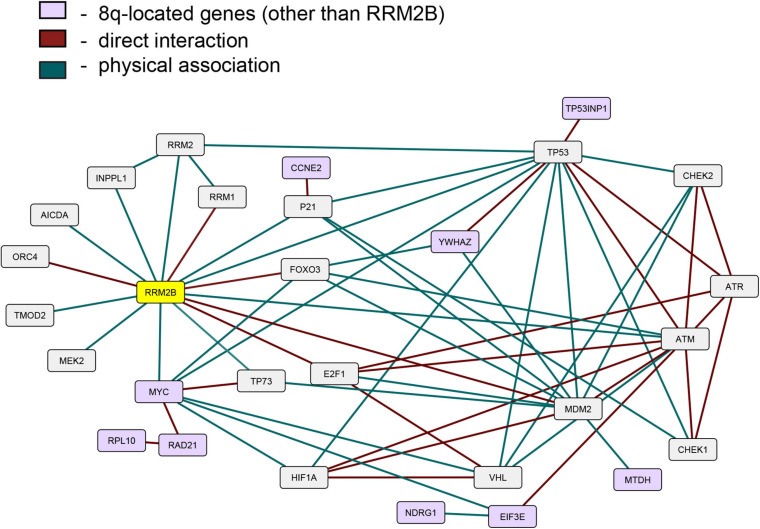
An interactive network of *RRM2B* and related genes. The network represents proteins that functionally interact or intersect with RRM2B (yellow) or other 8q-gene products (purple). Red lines represent direct interaction, and green lines—physical association (interactions classified according to the BioGRID database annotation).

Since the expression of *RRM2B*, *MYC*, and several other 8q-amplicon genes was increased, we next tested if the products of these genes within the 8q-amplicon interact with RRM2B. Figure 4 shows that RRM2B interacts with several proteins that are important for cell regulatory mechanisms such as DNA damage and response pathway, cell cycle, oxygen sensing and apoptosis. We also found that several 8q22–24 gene products (Figure 4, in light purple) also interact or intersect with the proteins in the RRM2B network. Finally, we performed a pathway enrichment analysis for all the interacting genes presented in Figure 4. Using WebGestalt tool (Liao et al., 2019), we found that the most significantly enriched pathways were signal transduction mediated by p53, response to DNA damage and other environmental stimuli, cell cycle checkpoints, DNA replication, response to oxygen levels and apoptotic signaling. The results are presented in Supplementary Table 3. Overall, these data suggest that amplifications in the 8q-region, observed in multiple cancers, may impact cancer cell survival due to their involvement and intersection in important cellular pathways.

### *RRM2B*-Amplified Tumors Exhibit Distinct Tumor Mutation Signatures

**FIGURE 5:**
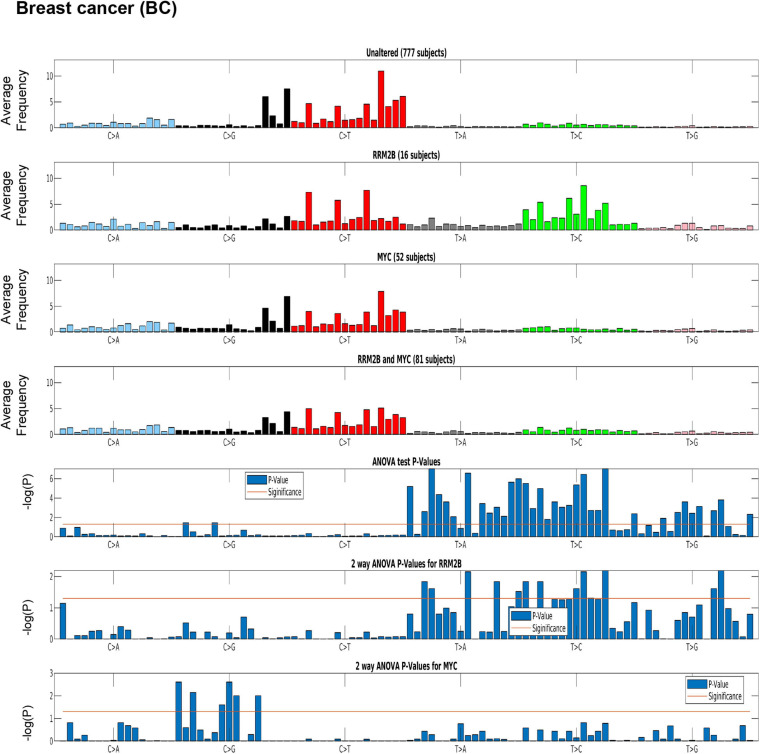
Mutation signature in breast cancer (BC) cases segregated by amplification type. One-way ANOVA (*RRM2B* amplifications only vs. other groups) and two-way ANOVA (included group with *RRM2B* and *MYC* co-amplifications) analysis showed that the T > C and T > G mutations are statistically significant. Top panels: tumor whole-exome sequence data from the PanCancer Atlas studies was used to calculate the average frequency of the 96 trinucleotide context mutations in each group: unaltered cases, cases with only *RRM2B* amplifications, cases with only *MYC* amplifications, and cases with amplifications in both. Bottom panels: The statistical significance of each comparisons is represented by ANOVA tests as -log10 (*P*-value) for each of the 96 trinucleotide context mutations. The -log10 (P) visualizations are provided for: one-way ANOVA comparing *RRM2B* only group to all other groups, a two-way ANOVA comparing all groups with *RRM2B* or *MYC* amplifications. -log10 (P)—the taller the bars are, the lower is the *P*-value and when the bars exceed the red line, *P-*values are less than 5%. *P-*values of each subfigure have been corrected using Benjamini–Hochberg procedure.

Distinct tumor mutation signatures have been associated with defects in certain genes or pathways, and with certain endogenous and exogenous exposures (Alexandrov et al., 2020). Here, we tested whether *RRM2B* amplifications are associated with specific tumor mutational signatures. For this analysis, we used the recent PanCancer Atlas data, reporting mutational spectra for 9493 individual tumors, including 926 BC, 384 OC, and 461 HNSCC and 524 LUAD (Alexandrov et al., 2020). Interestingly, BC cases with *RRM2B* amplification alone were significantly associated with T > C and T > A mutations (*P* < 0.05, Figure 5 and Supplementary Table 4).

A similar signature was observed in HNSCC, but not in OC (Supplementary Figure 5). The HNSCC and OC signatures could not be tested for significance as only few cases were present in the PanCancer Atlas data. For LUAD, a distinct signature of C > A mutations (significant by one-way ANOVA and two-way ANOVA tests as above, *p* < 0.05; Supplementary Figure 6 and Supplementary Table 4) was observed for cases with only *RRM2B* amplifications.

The observed mutation signatures for *RRM2B* amplifications were most similar to those that have been recently described for defects in distinct DNA replication and repair pathways (Alexandrov et al., 2020). The tumor mutation signatures observed have been associated with defective DNA base excision repair, and DNA mismatch repair. We also observed mutation signatures for presence of reactive oxygen species that can lead to extensive DNA base damage (Cadet and Wagner, 2013).

### *RRM2B* Amplifications Correlate With Clinical Outcome in ER + PR + HER2 + Breast Cancer

**FIGURE 6:**
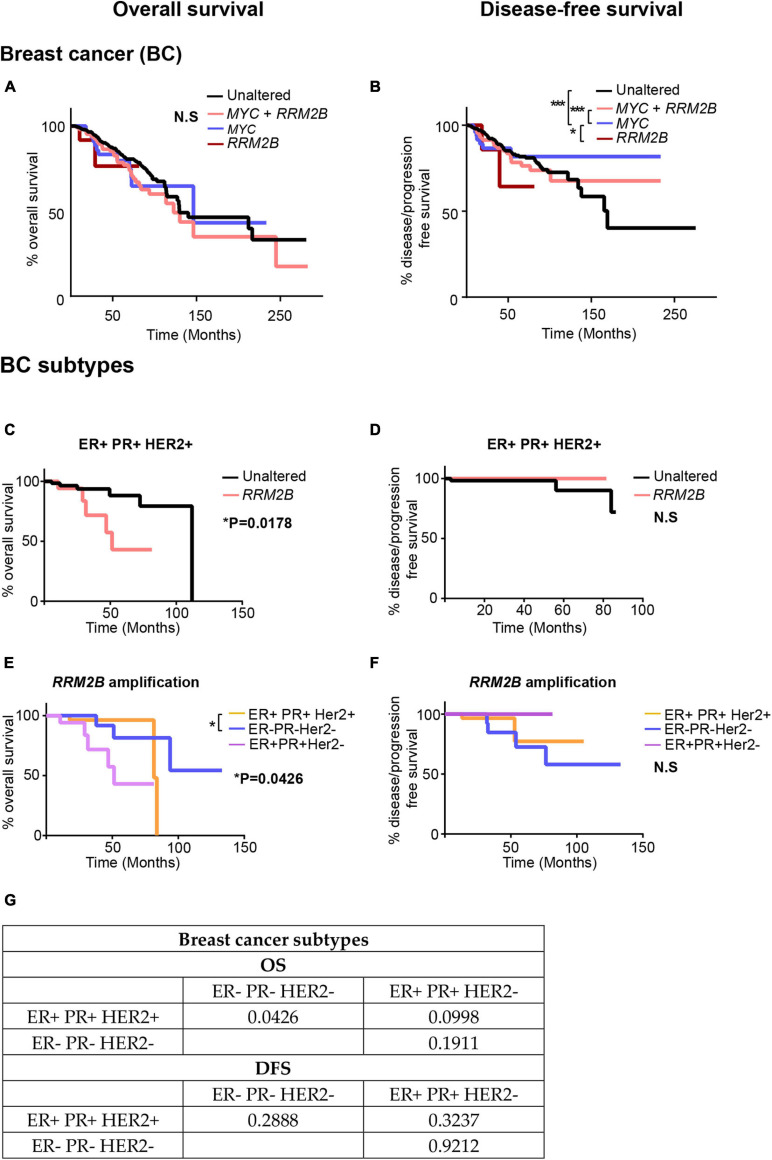
Kaplan-Meier curves for OS and DFS in breast cancer (BC) **(A,B)** and its subtypes **(C–G)** studies with *RRM2B* amplifications and/or *MYC* amplifications. **(A)** OS in BC cases. The cases that were unaltered for both genes (black, *n* = 737), cases with *RRM2B* amplifications (red, *n* = 14), cases with *MYC* amplifications (blue, *n* = 62) and co-amplifications (pink, *n* = 210) were plotted. **(B)** DFS in BC cases. The cases that were unaltered for both genes (black, *n* = 678), cases with *RRM2B* amplifications (red, *n* = 12), cases with *MYC* amplifications (blue, *n* = 55) and co-amplifications (pink, *n* = 184) were plotted. **(C)** OS in ER + PR + HER2 + BC cases. The cases that were unaltered for RRM2B (black, *n* = 68), cases with *RRM2B* amplifications (salmon, *n* = 18). **(D)** DFS in ER + PR + HER2 + cases. The cases that were unaltered for *RRM2B* (black, *n* = 63), cases with *RRM2B* amplifications (red, *n* = 13). **(E)** OS in BC subtypes with *RRM2B* amplifications. ER + PR + HER2 + cases with amplifications in *RRM2*B (purple, *n* = 18), ER- PR- HER2- cases with amplifications in *RRM2B* (blue, *n* = 26), and ER + PR + HER2- cases with amplifications in *RRM2B* (peach, *n* = 36). **(F)** DFS in BC subtypes with *RRM2B* amplifications. ER + PR + HER2 + cases with amplifications in *RRM2*B (purple, *n* = 13), ER- PR- HER2- cases with amplifications in *RRM2B* (blue, *n* = 25), and ER + PR + HER2- cases with amplifications in *RRM2B* (peach, *n* = 34). The plots were compared using Log-rank test and significance is shown as follows: *P < 0.05, **P < 0.01, and ***P < 0.001. **(G)** *P-*values for the graphs **(E,F)**.

Based on the distinct tumor signatures observed in *RRM2B*-amplified breast cancers (Figure 6), we next tested whether *RRM2B*-amplifications associate with clinical outcomes. In BC, no significant differences in OS were observed (Figure 6A), which is different from a previous finding in breast cancer (Chae et al., 2016). However, in comparison to OS, significant differences were observed for DFS (Figure 6B). *MYC* amplification alone had the best DFS, compared to *MYC* and *RRM2B* co-amplified cases, and unaltered cases (*p* < 0.0001). Additionally, *RRM2B* amplification cases tend to have worse DFS than *MYC* only cases (*p* = 0.0519).

To further explore the conflicting findings in BC, we analyzed *RRM2B* amplifications separately in each of the three major subtypes of breast cancer [ER + PR + HER2 + (*n* = 86), ER + PR + HER2- (*n* = 334), and triple negative ER-PR-HER2- (*n* = 100)]. We observed that patients with ER + PR + HER2 + BC bearing *RRM2B* amplifications had a significant decrease in OS (*p* = 0.0178, Figures 6C,E), with no difference in DFS (Figures 6D,F). Additionally, when comparing OS in all three BC subtypes with *RRM2B* amplifications, ER + PR + HER2 + patients (*n* = 18) had significantly worse OS than ER- PR- HER2- patients (*n* = 26) (*p* = 0.0426). The previous studies that observed worse OS in BC, based on *RRM2B* amplification, did not consider subtype differences, and may have only included the major BC subtype (ER + PR + HER2 +). Additionally, *p*-values for all comparisons (log-rank test and Gehan-Breslow-Wilcoxon test) in all the above BCs are available in Supplementary Tables 5, 6.

## Discussion

This study demonstrates that *RRM2B*, which is a major downstream target of p53, is highly amplified across multiple tumor types. Frequent *RRM2B* amplifications are a contrast to *TP53* loss of function mutations or deletions that are typically found in tumors. We found that *RRM2B*, which is present on chromosome 8, is usually co-amplified with *MYC* oncogene (which is also on chromosome 8). We also found, in multiple cancers, that tumors with co-amplified *RRM2B* and *MYC* exhibit significant amplification of the 8q22.3–8q24 region of chromosome 8. Intriguingly, we observed that several genes within the 8q-amplicon also interact with the RRM2B-functional network. Pathway enrichment analysis suggests the importance of 8q-region genes in response to oxygen levels, DNA replication, cell cycle, and DNA damage response.

We found that several gene products of the 8q22–24 region also interact or intersect with proteins in the RRM2B network. YWHAZ, an 8q-protein in the RRM2B network, binds and retains phosphorylated FOXO3 in the cytoplasm preventing its activity as a transcription factor, and apoptosis (Brunet et al., 1999). Under DNA damaging conditions, it has been shown that ATM-dependent activation of p53 leads to the formation of a binding site for YWHAZ, thus, increasing the affinity of p53 to bind with regulatory parts of cell cycle genes such as *CDKN1A*, *GADD45*, *MDM2* (Chernov and Gr, 1997). EIF3E, another 8q-protein in the RRM2B network, interacts with ATM and BRCA1 for the execution of DNA damage response and EIF3E alterations have been previously observed in breast cancer (Morris et al., 2012). These results suggest further investigation in cellular models to validate relationships between RRM2B, MYC and the identified interacting proteins. These data also suggest that amplifications in the 8q-region could have a profound effect on regulatory pathways such as DNA damage response and repair, cell cycle and oxygen sensing. It is well-appreciated that these regulatory pathways majorly impact response to cancer therapies and thereby clinical outcomes (Olcina et al., 2010; Luoto et al., 2013; O’Connor, 2015; Scanlon and Glazer, 2015; Ng et al., 2018; Begg and Tavassoli, 2020; Huang and Zhou, 2020); further warranting studies in cellular models of cancer.

The analysis of clinical outcomes in breast cancer revealed that based on the cancer subtype, co-alteration of *RRM2B* with *MYC*, or alone may significantly impact patient outcomes. The clinical implications of these data will be further illuminated by (a) a larger sample size of *RRM2B*-only amplified tumors and more balanced number of samples across each category, (b) analysis of outcomes in other cancers, and (c) other independent prognostic factors of overall survival. A previous study in breast cancer found frequent *MYC* co-amplifications with multiple genes in the 8q chromosomal region (Parris et al., 2014). This study concluded that these co-amplifications may explain the aggressive phenotypes of these tumors (Parris et al., 2014). *MYC* was found to be one of the most amplified genes in high-grade ovarian serous carcinomas, and the patients bearing *MYC-*amplified tumors had better overall survival (Macintyre et al., 2018). However, this study did not extend the analysis to other genes on chromosome 8. A recent study on therapeutic resistance in the highly heterogenous ovarian serous grade carcinomas consistently found amplifications in the 8q-region (starting from 8q22.3) in biopsies from different regions of the tumor, and the relapsed tumor tissue (Ballabio et al., 2019). These data suggest potential clinical significance of amplifications in the 8q-region. While *MYC* is the main driver for these tumors, the amplification *RRM2B* and the other 8q-genes may be relevant for cancer cell survival, with therapeutic implications.

Mutation signature analysis revealed that *RRM2B*-amplified tumors carry defects in distinct DNA repair pathways (base excision repair, DNA mismatch repair), and oxidative DNA damage. A previous study in mice found that overexpression of RRM genes combined with defective mismatch repair can lead to mutagenesis, and carcinogenesis (Xu et al., 2008). While a regulated expression of RRM2B reduces oxidative stress in a p53-dependent manner (Kuo et al., 2012), the impact of *RRM2B* overexpression on the levels of reactive oxygen species is not known. The observation of mutation signatures associated with increased reactive oxygen species suggests that *RRM2B* overexpression may impede the oxidative stress or oxygen sensing pathway. Finally, these observed mutation signatures suggest therapies that target defective DNA repair, such as PARP inhibitor therapy, may lead to clinical benefit in patients with *RRM2B*-amplified tumors (O’Connor, 2015; Nickoloff et al., 2017; Desai et al., 2018; Ma et al., 2018).

Alterations in genes involved in DNA replication and/or repair, and DNA damage response pathways are known to increase genetic instability, and lead to cancer (Chae et al., 2016). RRM2B is essential for DNA replication (nuclear and mitochondrial), DNA damage response and repair, protection from oxidative damage, and overall maintenance of genetic stability. Despite these known functions, the current understanding of RRM2B is limited to its role in MDS. Overall, in this study we provide an *in silico* analysis of *RRM2B* alterations in cancer and their potential significance. Further studies in cellular models are warranted to delineate the role of *RRM2B*, and other 8q-chromosome genes in cancer cell maintenance, therapeutic targeting and clinical outcomes.

## Conclusion

Overall, this study provides an in-depth analysis of *RRM2B* alterations in multiple tumor types, majorly reflected as *RRM2B* amplifications in conjunction with *MYC*, and other genes on chromosome 8. These cases exhibit a distinct 8q-amplification pattern as well as survival outcome differences and mutation signature differences, depending on cancer type and subtypes. Future genome-wide studies of other cancer datasets are warranted to confirm the results of the present study. Finally, studies in cellular models are required to delineate the role of *RRM2B*, and other 8q-chromosome genes in cancer cell maintenance, therapeutic targeting and clinical outcomes.

## Data Availability Statement

Publicly available datasets were analyzed in this study. This data can be found here: www.cbioportal.org.

## Author Contributions

WI and ED: methodology, formal analysis, investigation, roles, writing—original draft, data curation, visualization, and validation. SS: formal analysis, investigation, roles, writing—original draft, and data curation. TV: software, formal analysis, and data curation, and visualization. GR: writing—review and editing, methodology, supervision, and funding acquisition. SA: conceptualization, methodology, writing—review and editing, supervision, project administration, resources, and funding acquisition. All authors contributed to the article and approved the submitted version.

## Conflict of Interest

The authors declare that the research was conducted in the absence of any commercial or financial relationships that could be construed as a potential conflict of interest.

## Footnotes

1 https://useast.ensembl.org/

2 http://www.cbioportal.org

3 synapse.org

4 http://www.cancerindex.org
