# Supplementary material for: RRM2B Is Frequently Amplified Across Multiple Tumor Types: Implications for DNA Repair, Cellular Survival, and Cancer Therapy
Source: Front Genet. 2021 Mar 12;12:628758. doi: 10.3389/fgene.2021.628758 (PMC8045241; doi:10.3389/fgene.2021.628758)
Supplement: Supplementary file 1 [file Data_Sheet_1.PDF]

Supplementary figures

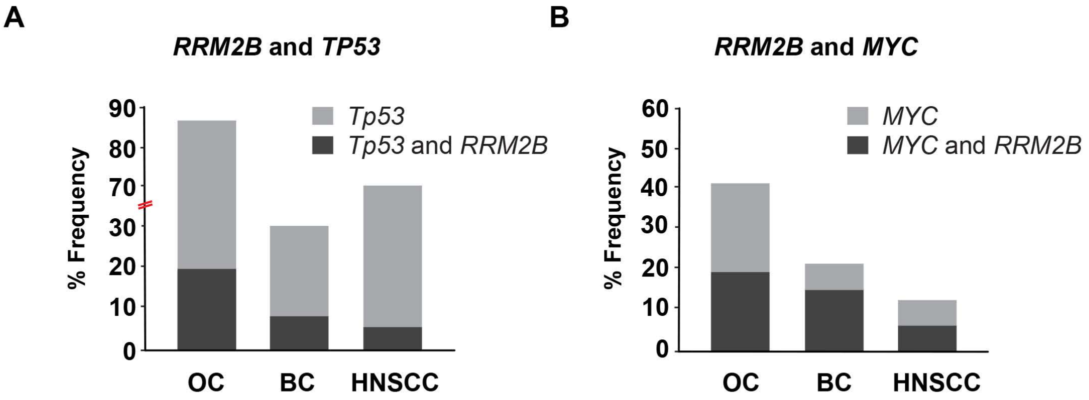

Supplementary Figure 1. Somatic alteration frequencies in RRM2B along with either MYC or Tp53 in OC, BC and HNSCC. A: Comparison of frequency between cases with both RRM2B and Tp53 alterations (dark gray) and cases with Tp53 alterations alone (light gray). B: Comparison of frequency between cases with both RRM2B and MYC alterations (dark gray) and cases with MYC alterations alone (light gray).

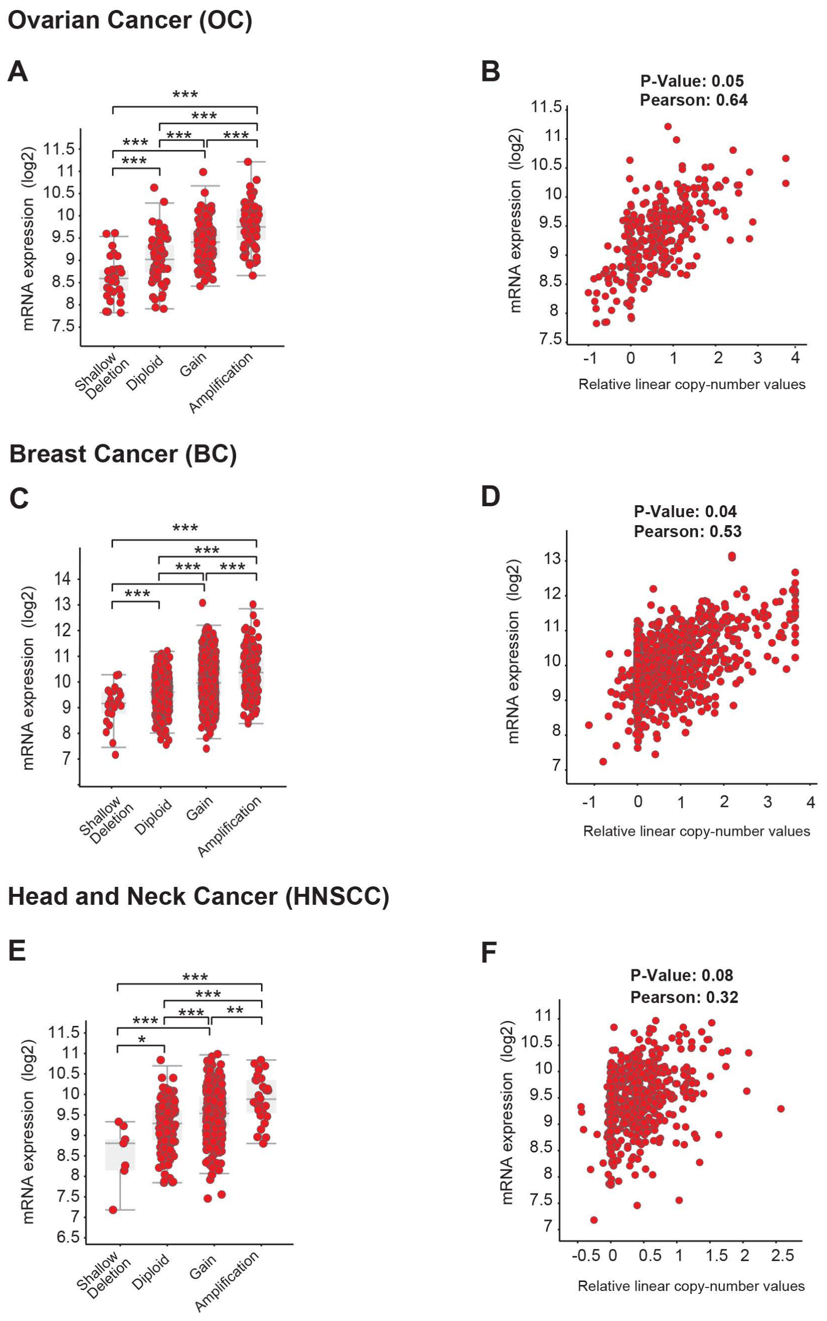

Supplementary figure 2. *RRM2B* mRNA expression based on copy number alterations in ovarian (OC), breast (BC) and head and neck (HNSCC) cancers. A, C, E: log2 mRNA expression values from cases with different copy number alterations (shallow deletion to amplifications). Mann-Whitney Non-Parametric T-tests were performed on the data and differences between the alteration types is represented by P-value *<0.05, ** for P-value <0.01 and *** for P-value <0.001. B, D, F: log2 mRNA expression values based on relative copy number alterations in *RRM2B*. The Pearson correlation values (for correlation between increase in mRNA expression and increase in relative copy number values) are listed for each comparison.

Breast Cancer (BC)

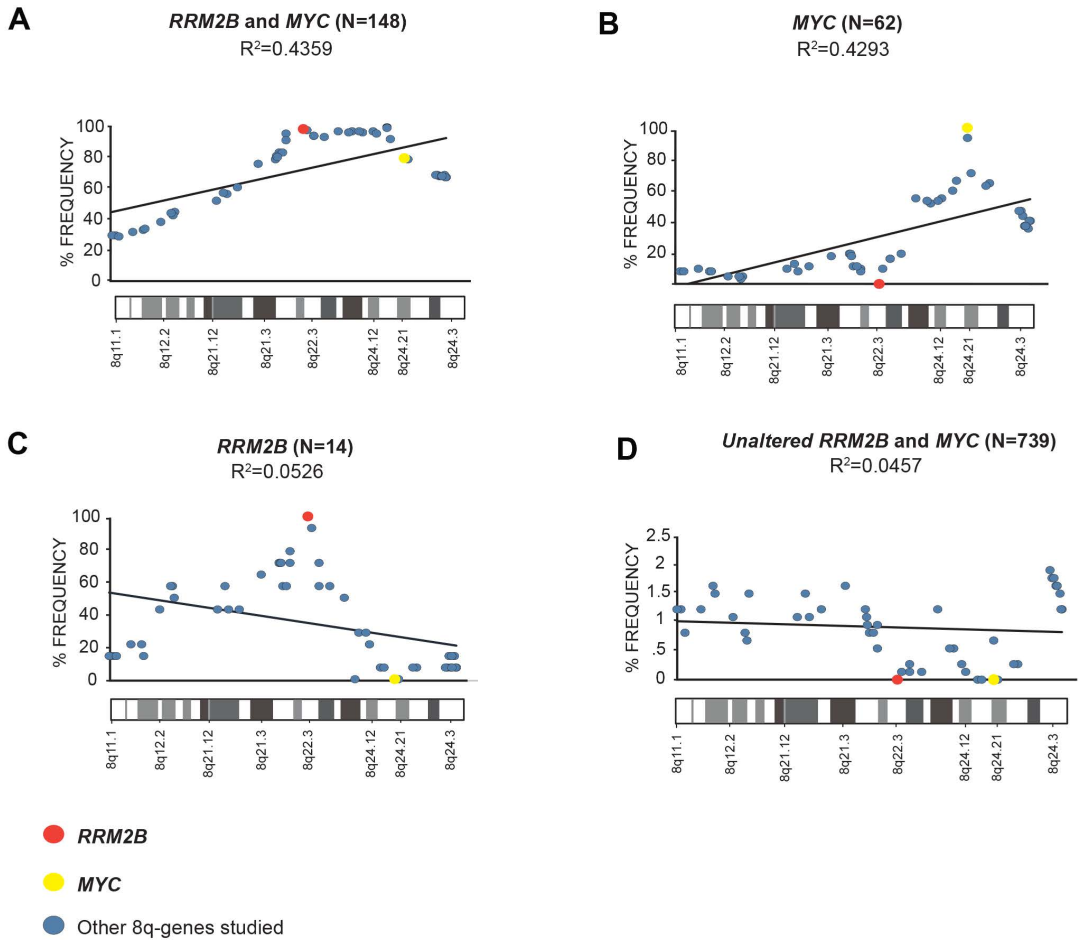

Supplementary Figure 3. Amplification frequencies of 8q-genes in breast cancer (BC) study. A: cases with co-amplification of RRM2B and MYC. B: cases MYC only amplification. C: cases RRM2B only amplifications. D: cases with neither (unaltered) were plotted as percent of frequency for amplifications in various 8q-region genes relevant for cancer (see Supplementary Table 1). RRM2B (red circle), MYC (yellow circle), and other genes (blue circle). The Pearson correlation (R^2 value) for the data points is represented by a black trend line.

Head and Neck Cancer (HNSCC)

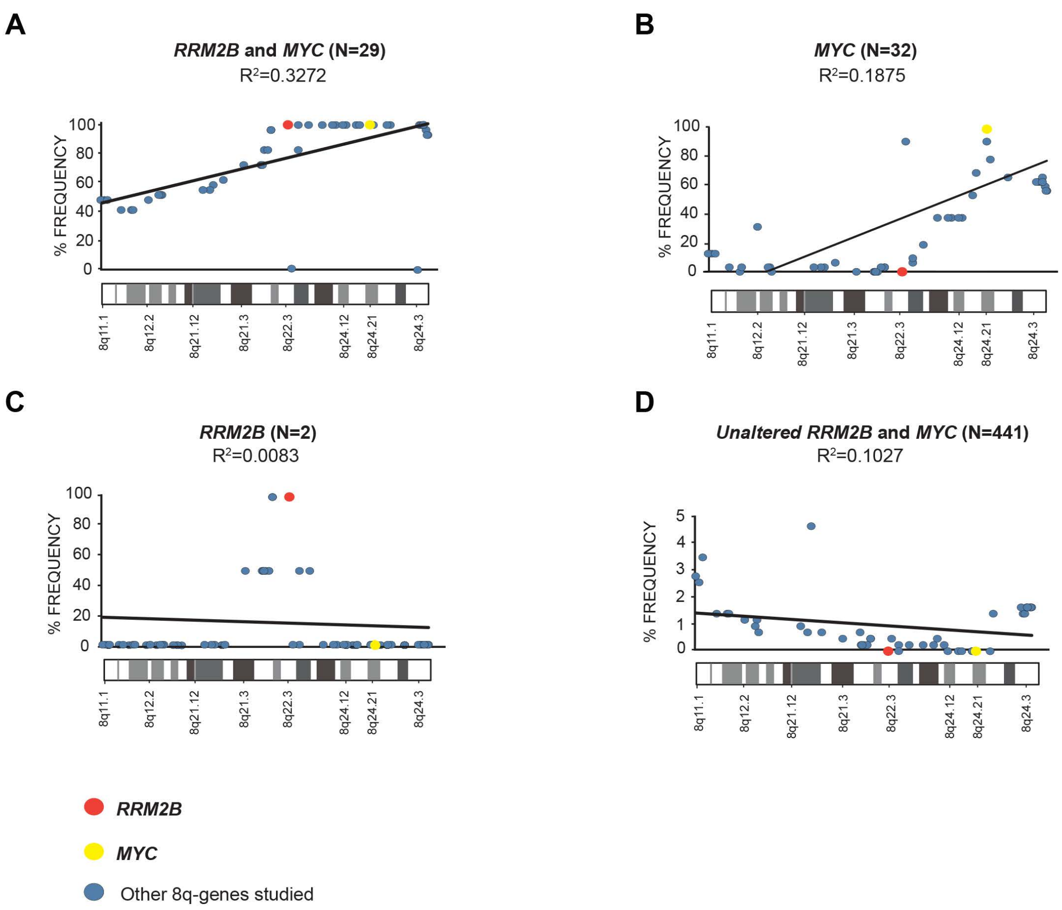

Supplementary Figure 4. Amplification frequencies of 8q-genes in head and neck cancer (HNSCC) study. A: cases with co-amplification of RRM2B and MYC. B: cases MYC only amplification. C: cases RRM2B only amplifications. D: cases with neither (unaltered) were plotted as percent of frequency for amplifications in various 8q-region genes relevant for cancer (see Supplementary Table 1). RRM2B (red circle), MYC (yellow circle), and other genes (blue circle). The Pearson correlation (R^2 value) for the data points is represented by a black trend line.

Ovarian cancer (OC)

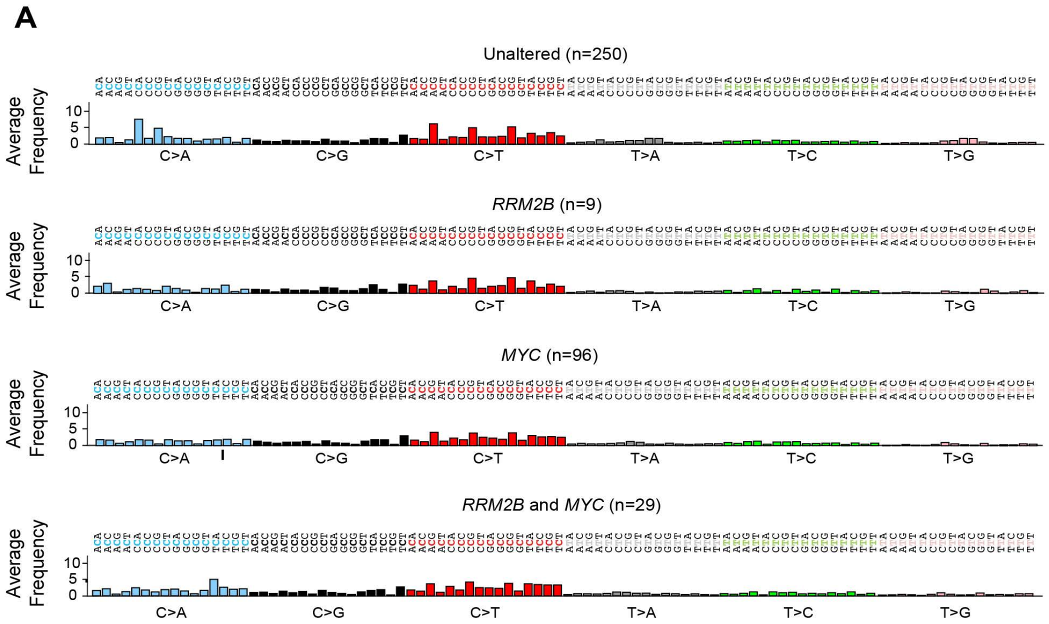

Head and Neck cancer (HNSCC)

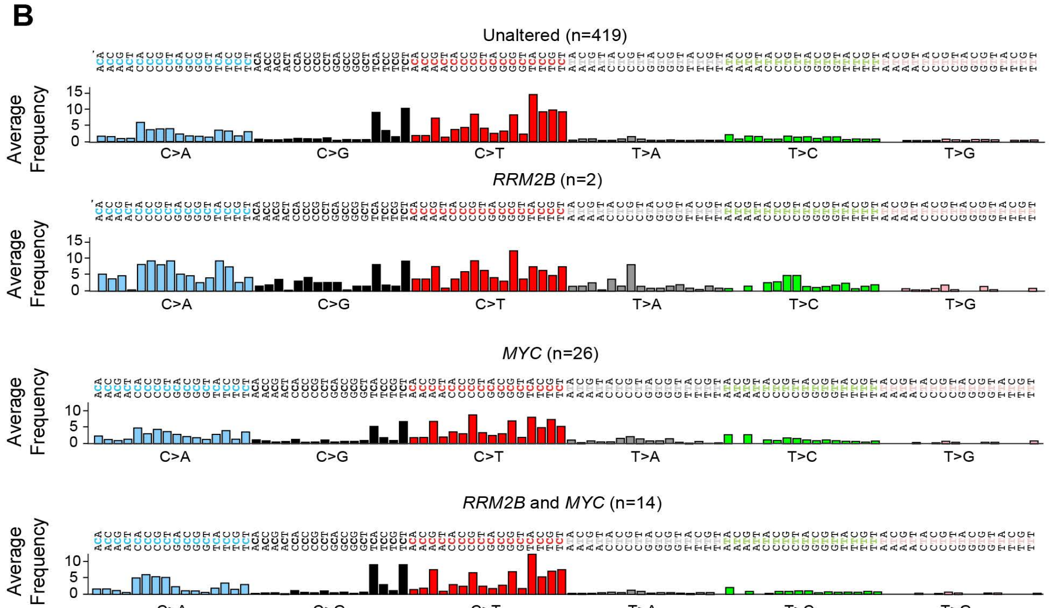

Supplementary Figure 5. Mutation signatures of OC and HNSCC cancers based on RRM2B or MYC amplifications. Tumor whole-exome sequence data from the PanCancer Atlas studies was used to calculate the average frequency of the 96 trinucleotide context mutations in each group: unaltered cases, cases with RRM2B or MYC amplifications only, and cases with both. A: Mutation Signatures in OC cancer. B: Mutation Signatures in HNSCC.

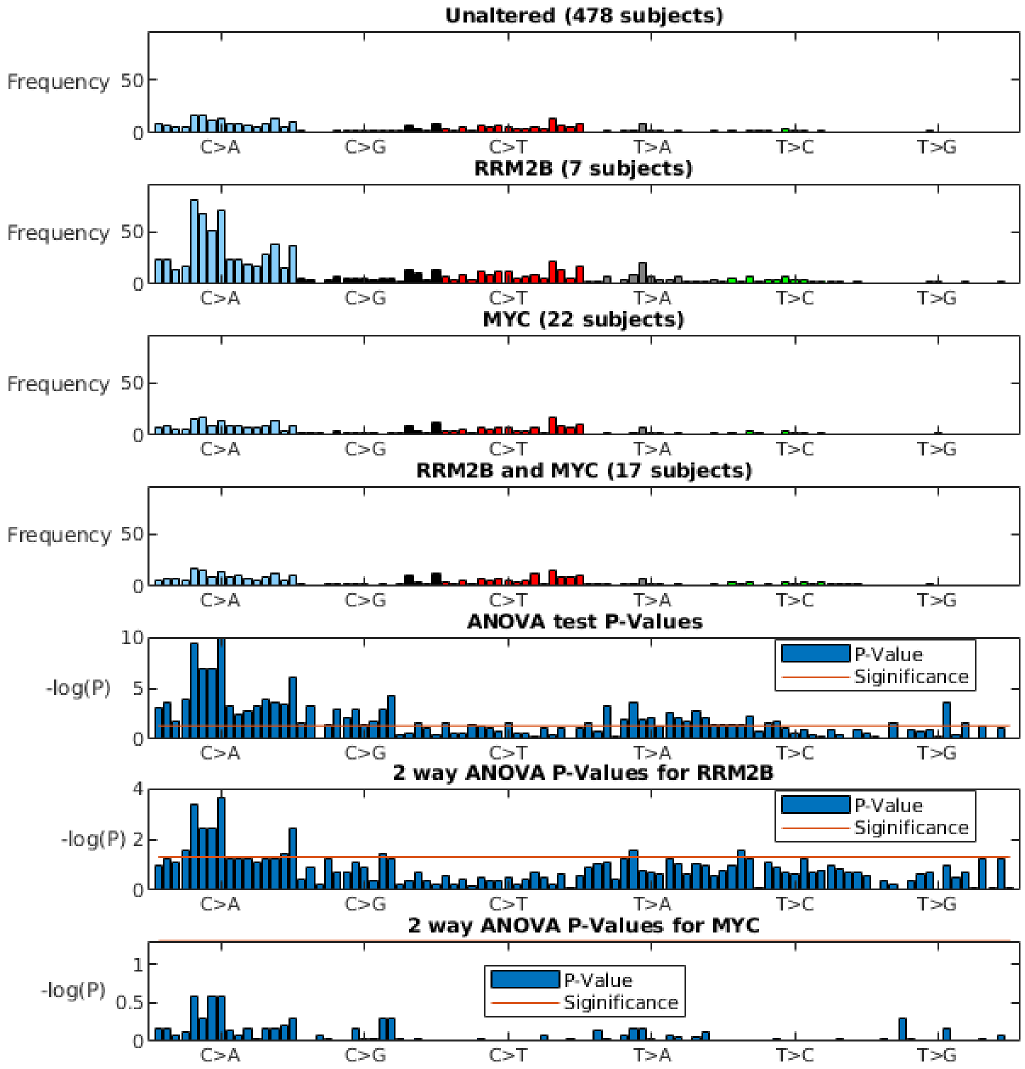

Supplementary Figure 6. Mutation signature of lung cancer (LUAD) patients based on *RRM2B* or *MYC* amplifications. A one-way ANOVA (*RRM2B* amplifications only versus other groups) and two-way ANOVA (included group with *RRM2B* and *MYC* co-amplifications) analysis showed significant C>A mutations. Top: Tumor whole-exome sequence data from the PanCancer Atlas studies was used to calculate the average frequency of the 96 trinucleotide context mutations in each group: unaltered cases, cases with *RRM2B* or *MYC* amplifications only, and cases with both. Bottom: The statistical significance of each comparison is represented by an inverse transformed p-value, $-\log_{10}$ (P-value), calculated by an ANOVA test on each group of signatures (96 trinucleotide context mutations) compared. The $-\log_{10}$ (P) results are provided for: one-way ANOVA comparing the *RRM2B*-only group to the other groups (panel 1) and a two-way ANOVA comparing all groups with *RRM2B* (panel 2) or *MYC* amplifications (panel 3). Since -log10 (P) is employed here, longer bars correspond to smaller P-Values, with bars above the red line being P-values less than 0.05. P-values of each subfigure have been corrected using Benjamini–Hochberg procedure.
